# The water use of Indian diets and socio-demographic factors related to dietary blue water footprint

**DOI:** 10.1016/j.scitotenv.2017.02.085

**Published:** 2017-06-01

**Authors:** Francesca Harris, Rosemary F Green, Edward J M Joy, Benjamin Kayatz, Andy Haines, Alan D Dangour

**Affiliations:** a Department of Population Health, London School of Hygiene & Tropical Medicine, Keppel Street, London WC1E 7HT, UK; b Leverhulme Centre for Integrative Research on Agriculture and Health (LCIRAH), 36 Gordon Square, London WC1H 0PD, UK; c GFZ German Research Centre for Geosciences, Telegrafenberg, 14473 Potsdam, Germany; d Department of Social and Environmental Health Research, London School of Hygiene & Tropical Medicine, 15-17 Tavistock Place, London WC1H 9SH, UK

**Keywords:** WF, water footprint, IMS, Indian Migration Study, FFQ, food frequency questionnaire, SLI, standard of living index, NSSO, National Sample Survey Organisation, WHO, World Health Organization, CI, confidence interval, SD, standard deviation, Food consumption, Sustainability, India, Freshwater use

## Abstract

Agriculture accounts for ~ 90% of India's fresh water use, and there are concerns that future food production will be threatened by insufficient water supply of adequate quality. This study aimed to quantify the water required in the production of diets in India using the water footprint (WF) assessment method. The socio-demographic associations of dietary WFs were explored using mixed effects regression models with a particular focus on blue (irrigation) WF given the importance for Indian agriculture. Dietary data from ~ 7000 adults living in India were matched to India-specific WF data for food groups to quantify the blue and green (rainfall) WF of typical diets. The mean blue and green WF of diets was 737 l/capita/day and 2531 l/capita/day, respectively. Vegetables had the lowest WFs per unit mass of product, while roots/tubers had the lowest WFs per unit dietary energy. Poultry products had the greatest blue WFs. Wheat and rice contributed 31% and 19% of the dietary blue WF respectively. Vegetable oils were the highest contributor to dietary green WF. Regional variation in dietary choices meant large differences in dietary blue WFs, whereby northern diets had nearly 1.5 times greater blue WFs than southern diets. Urban diets had a higher blue WF than rural diets, and a higher standard of living was associated with larger dietary blue WFs. This study provides a novel perspective on the WF of diets in India using individual-level dietary data, and demonstrates important variability in WFs due to different food consumption patterns and socio-demographic characteristics. Future dietary shifts towards patterns currently consumed by individuals in higher income groups, would likely increase irrigation requirements putting substantial pressure on India's water resources.

## Highlights

• First analysis of the water used in the production of diets in India using individual-level food consumption data.
• The dietary blue (irrigation) water footprint in India is greater than estimates from high-income countries.
• Geographic region and socio-demographic factors are strongly associated with dietary blue water footprint.

## Graphical abstract

**Figure:**
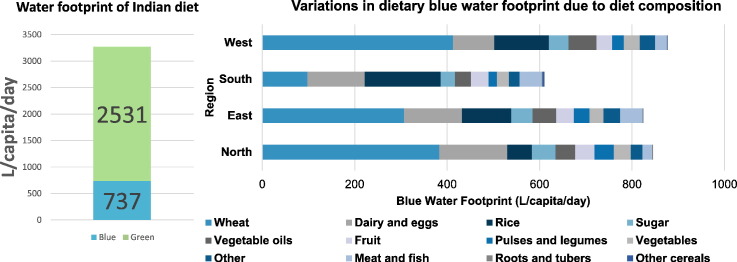

## 1 Introduction

Growing populations and changing food consumption patterns are placing increased pressure on natural and agricultural systems. To ensure sustainable and healthy food systems, a combination of consumption- and production-side changes will be required (FAO, 2010, Smith et al., 2008). Many studies assessing environmental impacts of diets have focused on greenhouse gas emissions, largely in high income settings (Berners-Lee et al., 2012, Jones et al., 2016, Macdiarmid et al., 2012, Pathak et al., 2010). However, much less evidence is available on the water use associated with the production of diets which remains a major sustainability issue as agriculture accounts for ~ 70% of global water withdrawals (FAO, 2016). Dietary water use can be quantified using the water footprint (WF) concept (Cazcarro et al., 2012, Renault and Wallender, 2000, Vanham, 2013, Vanham et al., 2013). The WF Network has estimated the WF of crops and crop-derived products through a globally-gridded, multi-layer dataset (Mekonnen and Hoekstra, 2011), and used this to calculate the WFs of national food supplies (Hoekstra and Mekonnen, 2012). The WF is divided into three parts: green, blue and grey. For crops, the green WF represents the volume of precipitation expended during production, calculated from total rainwater evapotranspiration plus the water incorporated into the harvested crop. The blue WF represents the volume of ground and surface water delivered to crops through irrigation (Hess et al., 2015). The grey WF represents the volume of freshwater that would be required to dilute agricultural pollution to meet water quality standards (Aldaya et al., 2012). For livestock, WFs are derived from feed crop WFs and drinking and service water.

Analysing the water use associated with diets is particularly important for India, where ~ 90% of water withdrawal (ground and surface water use) is used for irrigated agriculture, making India the largest user of groundwater in the world (FAO, 2016). However groundwater resources are depleting in many areas (Rodell et al., 2009), particularly in the Indo-Gangetic Basin where the rice-wheat double cropping system is widely practiced (Hoekstra et al., 2012, Tiwari et al., 2009). Additionally, future environmental change could have implications for Indian agriculture. Predicted increases in temperature (Dash et al., 2007, Salvi and Ghosh, 2013) could reduce crop yields and water-use efficiency of rice and wheat (Jalota et al., 2013), although evidence remains conflicting (Deryng et al., 2016). Changes to melt water may reduce flow to key river basins for India's groundwater (Immerzeel et al., 2010), so water scarcity could worsen (Gerten et al., 2011). Furthermore, uncertainty in future rainfall patterns (Salvi and Ghosh, 2013) means ground and surface water resources may become an even more important irrigation reserve.

Quantifying the WF of diets and assessing socio-demographic drivers provides a valuable consumption-side perspective on water resource use and can inform strategies to improve the sustainability of the food system. For example, the approach may help to forecast the potential implications of dietary change on water resource use. The aims of this study were two-fold: first, to quantify the green and blue WFs of typical Indian diets by matching dietary data from a large cross-sectional study of Indian adults to green and blue WFs of food items. Grey WFs are not considered in this study. Due to the importance of the local climate and environment on crop water use, spatial variations in WFs were explored for the two major cereals, rice and wheat. Secondly, to explore the socio-demographic factors associated with dietary blue WF. Blue WFs, although typically much smaller than green WFs, are a particular concern given India's agricultural production is highly dependent on irrigation and availability of groundwater is a significant current issue.

## 2 Methods

### 2.1 Socio-demographic characteristics and dietary data

Dietary and population data were derived from the Indian Migration Study (IMS) as it provides in-depth data for > 7000 Indian adults. The IMS was conducted during 2005–2007 as part of a pre-existing screening study of cardiovascular disease risk factors among Indian adults. The study used a cross-sectional sib-pair design to study factory workers who had migrated to one of the four following Indian cities – Bangalore, Hyderabad, Lucknow and Nagpur, and their rural-dwelling siblings and co-resident spouses. A 25% sample of urban non-migrants was also recruited. A total of 7067 individuals were included in the final sample with 90% of rural participants and 98% of urban participants living in four states (Karnataka, Andhra Pradesh, Maharashtra and Uttar Pradesh). Full details of the sampling methodology and study design have been reported elsewhere (Ebrahim et al., 2010, Lyngdoh et al., 2006).

Dietary intake was measured through an interviewer-administered semi-quantitative food frequency questionnaire (FFQ), assessing consumption of 199 common food items. For the current analysis, the 199 items were aggregated into 36 food groups based on similarity in nutritional content (Appendix Table A.1; Joy et al., accepted for publication). Reliability of the FFQ was assessed by selecting a subsample to repeat the questionnaire 1–2 months (n = 185), and 12 months (n = 305) after completion during the original period of data collection. A further 530 participants carried out three 24 h recalls as a reference method used to validate the FFQ. Most food items yielded acceptable validity (Ebrahim et al., 2010). However, to reduce the sensitivity of WF estimates to dietary intake reporting error, participants with extreme values for dietary energy intakes (mean ± 2 ∗ SD) were excluded (n = 292).

Information on socio-demographic characteristics was obtained through an interviewer-administered questionnaire. A Standard of Living Index (SLI) was calculated using an asset-based survey on 14 items, including quality of house, toilet facilities, land ownership, and source of lighting.

### 2.2 Water footprints of food items

The WF Network has quantified the WF of crops using a grid-based dynamic water balance model that considers local climate, soil factors, and rates of nitrogen fertiliser use (Mekonnen and Hoekstra, 2011). At the time of study, the majority of food consumed in India was produced domestically with little contribution from imports (FAOSTAT, 2016), so India-specific WF data were used. Due to the large size and varied environment of India, WFs of typical food items are reported at state-level, with green and blue WFs (l/g of food) available for the years 1996–2005 (www.waterfootprint.org; Mekonnen and Hoekstra, 2011). For animal products, WFs are reported by production system (i.e. industrial, grazing or mixed) at a national level, based on the volume and composition of feed, drinking and service water use, and conversion to edible product (Mekonnen and Hoekstra, 2012).

For the present study, state-level WFs of animal products were quantified based on methods from Mekonnen and Hoekstra (2012). Briefly, seven farm categories were considered: beef cattle, dairy cattle, pig, sheep, goat, broiler chicken and layer chicken. The WF of each category was estimated from the indirect WF of feed using state-level crop WFs and the direct water consumption from drinking and services. The volume and composition of feed was calculated for each animal category under grazing, industrial and mixed production systems (Mekonnen and Hoekstra, 2012), with their relative occurrence in India used to obtain a weighted average for each state. Feed conversion efficiencies in South Asia region and the drinking and service water use for each animal category were derived from Mekonnen and Hoekstra (2012).

The WF Network database does not report national WFs of seafood products. The majority of the IMS population was situated away from coastlines, and the total intake of seafood (fish and prawns), was small. Therefore, we chose to calculate the WFs based on the WF of fish feed with the assumption that all fish were farmed (Pahlow et al., 2015). Carp accounts for > 90% of freshwater fish production in India so the WF of fish was based on major carp species. The volume of commercial feed in India was calculated using annual production figures of the major species (Suresh, 2007), feed conversion ratios, and the fraction of commercial feed in total feed (Pahlow et al., 2015). Data on the composition of feed were obtained from Suresh (2007) and FAO AFFRIS (FAO, 2016).

### 2.3 Matching dietary intake and WF datasets

The 199 IMS food items were matched to products in the WF Network database using author judgement (see Appendix Table A.1). Items were excluded from the analysis if there was no suitable match or they represented < 5% of the food groups' consumption (total of 33 out of 398 across green and blue WF data points; see Appendix Table A.1). The WFs of the 36 food groups were calculated from the mean WF of constituent items, weighted by the relative contribution of food items to total food group consumption across the IMS population. The majority of IMS participants resided in urban areas and the location of consumption was unlikely to give a good prediction of the location of production. Thus, in the absence of comprehensive data on interstate trade of major food items, we used a national average value for the WFs of the 36 food groups. However, we maintained a framework that allowed us to investigate spatial variations in WF values and thus the uncertainty of our estimates. Therefore, the WFs of food groups were initially derived at state level and a mean national-level WF for each food group was calculated by weighting the state values by land size (Source: Office of Registrar General of India, Ministry of Home Affairs, see Appendix Table A.2). The justification for this approach (rather than simply using national-level WFs reported by the WF Network) is considered in the Discussion section.

### 2.4 Quantifying and analysing the WF of individual diets

The blue and green WFs of IMS participants' diets were quantified by combining individuals' mean daily consumption of each food group with the matched national-weighted WF data. The socio-demographic characteristics assessed for their relationships with dietary blue WF were age, gender, region, rural/urban residency, religion, SLI score and education (Table 1). Spearman's rank correlation matrix was used to assess bivariate relationships between predictor variables, and those identified as very strongly correlated (*R* > 0.7) were excluded prior to analysis. The crude associations between socio-demographic characteristics (predictors) and the blue WF of diets (outcome) were assessed using separate mixed-effects linear regression models. Due to the sib-pair clustered design, each model specified the between-pair variation as a family-specific random effect in addition to the fixed effects used for each predictor variable. The reference category was chosen either as the most numerous or the lowest for ordered categorical variables. A combined mixed-effect multiple regression model was used to assess the adjusted relationships with all socio-demographic variables simultaneously. For comparison, each variable was assessed with the same multivariate model, containing each predictor and occupation as a confounder. The variance inflation factor (VIF) was used to assess multicollinearity as a multiple linear regression model. To assess the effect of diet composition (rather than total energy content of the diet) on blue WF, separate analysis was carried out adjusting for total calorie intake in the multivariate model. All statistical analyses were conducted using STATA (v.14; StataCorp LP, Texas, USA).

### 2.5 Ethical approval

Ethical approval for the IMS was obtained from the All India Institute of Medical Sciences Ethics Committee, reference number A-60/4/8/2004. The present study forms part of the Sustainable and Healthy Diets in India (SAHDI) project which was granted ethical approval by the London School of Hygiene & Topical Medicine (reference number 11509).

## 3 Results

The analysis included a total of 6775 participants, aged 17–76 years (mean 41 years). The sample contained more males (57.3%) than females, and the majority of participants were Hindu (91.1%). Descriptive characteristics of the study population are shown in Table 1.

### 3.1 Diet characteristics

Detailed dietary characteristics of the IMS population have been reported elsewhere (Bowen et al., 2011). The mean ± SD total dietary energy of the diets was 2883 ± 833 kcal/capita/day. Characteristics of the diet are shown in Table 2. The main staple cereals were rice and wheat, but large regional differences existed: southern diets had the highest mean consumption of rice (229 g/capita/day) and lowest of wheat (71 g/capita/day), whereas northern diets had the highest consumption of wheat (281 g/capita/day) and lowest of rice (74 g/capita/day). Males consumed more than females for each food category. Meat consumption was generally low but was higher in the south and east, and higher among non-Hindus. Fruit and vegetable, and dairy and eggs consumption increased with higher standard of living index (SLI).

### 3.2 Green and blue water footprints of foods groups and diets in India

#### 3.2.1 The water footprints of diets in India

The mean ± SD blue WF of diets in the study population was 737 ± 263 l/capita/day, and the mean ± SD green WF of diets was 2531 ± 885 l/capita/day (Table 3). Rice and wheat were the highest contributors to the dietary blue WF, consistent with their high proportion in the diet. Vegetable oils were the highest contributor to dietary green WF. Fruits and vegetables shared < 10% of both dietary blue and green WFs. Based on the average diet, wheat contributed 30.9% of the dietary blue WF while comprising 20.4% of total dietary energy, while rice contributed 18.7% and comprised 21.4% of total dietary energy.

#### 3.2.2 Water footprint of food groups in India

**Fig. 1:**
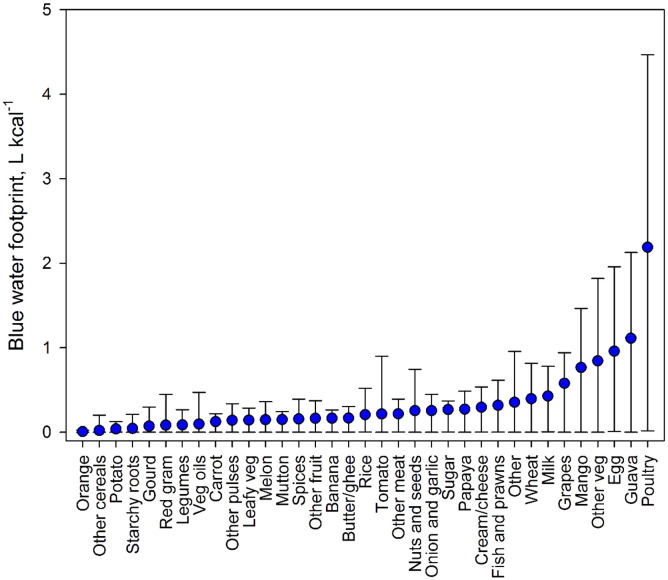
The blue water footprint (WF) of 36 food groups in India. Bars indicate the range of state-level values (min to max).

**Fig. 2:**
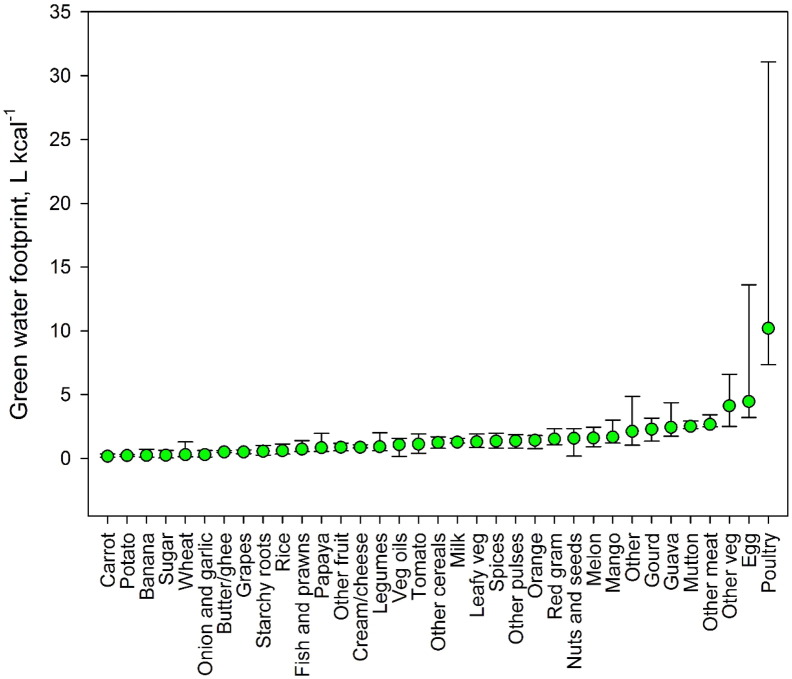
The green water footprint (WF) of 36 food groups in India. Bars indicate the range of state-level values (min to max).

There were large differences between the WF of food items in India (see Appendix Table A.3 for weighted averages of the 36 food items). Based on the weighted average WF across states, food items with the greatest green and blue WF per g of product were nuts and seeds, poultry and milk products. Wheat had a greater blue WF than rice (i.e. 1.37 l/g vs 0.72 l/g) but a lower green WF (i.e. 0.98 l/g vs 2.07 l/g). The blue WF of fruits and vegetables was relatively low, i.e. 0.25 l/g and 0.09 l/g respectively. However, when considering WFs per kilocalorie, the relationships altered (Fig. 1, Fig. 2). For some fruits, such as mango and guava, the WF greatly increased relative to other food items. This reflects the low energy density of food items with high moisture content. Wheat still had a higher blue WF per kilocalorie compared to rice, i.e. 0.4 l/kcal compared to 0.2 l/kcal. Poultry and animal products remained high for both green and blue WF.

**Fig. 3:**
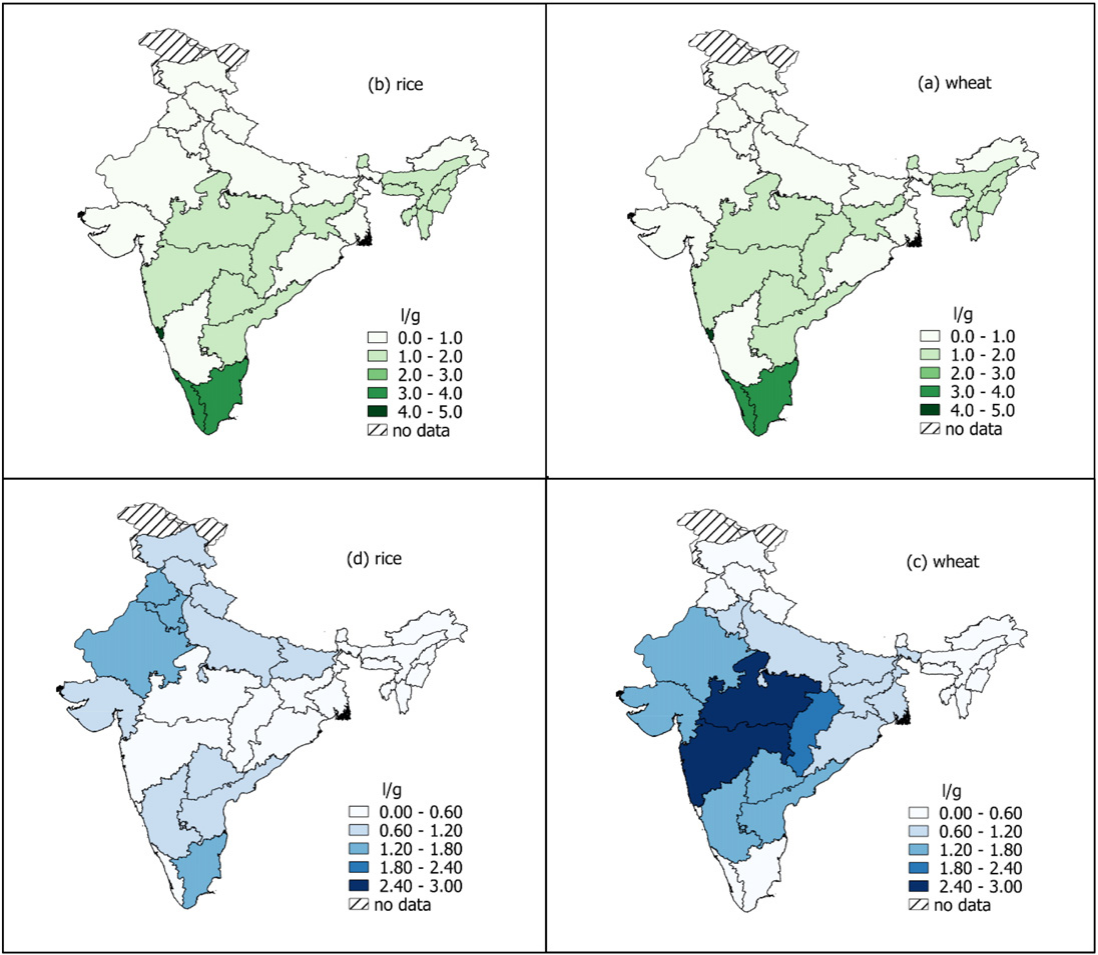
The green and blue water footprints of wheat (panels a and c) and rice (panels b and d) by state in India. Water footprint data are from the Water Footprint Network (Mekonnen and Hoekstra, 2011); boundary polygons were downloaded from the GADM database of Global Administrative Areas (version 2.8, http://www.gadm.org/) and Natural Earth Data (http://www.naturalearthdata.com). Mapping software: QGIS version 2.20.1.

The WF of food items varied greatly between states. The blue WF of wheat was highest in the central/western states, whereas the blue WF of rice was highest in north-western and south-eastern states (Fig. 3). The green WF of wheat was highest in the most southern states, whereas rice was highest in central states. Additionally, the green and blue WFs were negatively correlated for some states (Fig. 3). This reflects the methodology for quantifying blue WFs, whereby the water requirements of crops are assumed to be met by irrigation if sufficient precipitation or soil moisture is not available (Mekonnen and Hoekstra, 2011). Differences could also be seen for livestock WF where the mean blue WF of poultry products was 2.2 l/kcal, ranging from 4.5 l/kcal in Rajasthan to 0.007 l/kcal in Mizoram (Fig. 1). In this study, the variability of the WF of animal products relates to the variability in the WF of the feed ingredients and a low blue WF indicates that the feed crops were likely grown with minimal irrigation inputs.

### 3.3 Associations between dietary blue water footprint and socio-demographic characteristics

**Fig. 4:**
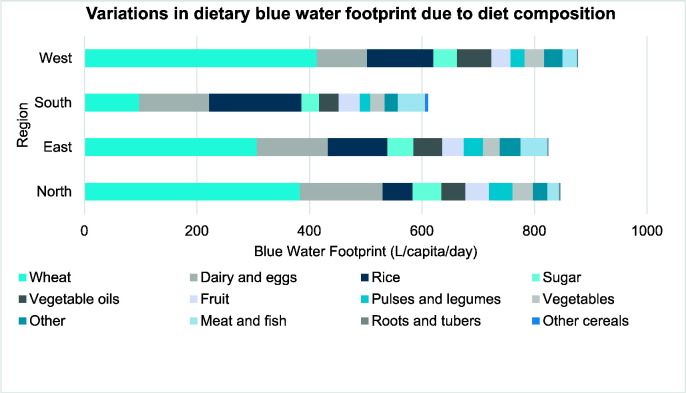
The regional variation in dietary blue water footprint in India due to diet composition. National-weighted WF figures are used.

There were large regional differences in dietary patterns that resulted in substantially different blue WFs (Fig. 4). Southern diets had the lowest overall blue WF (Table 4), mainly related to lower wheat consumption. Meat consumption was higher in the south and east but this did not result in a substantially higher overall blue WF because the quantities of meat consumed were small.

A total of seven socio-demographic characteristics were assessed for their relationship with dietary blue WF (Table 4). The unadjusted analysis showed that blue WF was significantly associated with all variables analysed.

After adjusting for socio-demographic confounders, there was strong evidence that age was independently and negatively associated with dietary blue WF (Table 4). The blue WF of diets was also associated with gender, with females lower than males (*R* = − 140 l/capita/day, 95% CI − 130 to − 151 l/capita/day, p < 0.001). Region was a strong predictor of dietary blue WF, and showed the largest effect size between the groups compared to the other socio-demographic variables. Dietary blue WF was lowest in the south and highest in the western regions (compared to south, *R* = 212 l/capita/day, 95% CI 194–230 l/capita/day, p < 0.001). Rural participants had a lower dietary blue WF compared to urban, and Hindus' dietary blue WF was lower than other religions. Socio-economic indicators were associated, with blue WFs increasing with higher levels of formal education and increasing SLI.

Additional adjustment for total dietary energy intakes attenuated many of these relationships, indicating that both the amount and the type of food consumed were associated with dietary blue WF (Table 4). The relationship with region remained similar, hence southern diets still had the lowest blue WF, although northern participants now had the highest dietary blue WF (*R* = 110 l/capita/day, 95% CI 104–117 l/capita/day, *p* < 0.001). Other socio-demographic factors including age, sex, location of residence and standard of living index remained associated with blue WF but effect sizes were modest. Adjusting for energy strengthened the evidence that people from other religions had a higher diet WF than Hindus, suggesting that diet pattern rather than total food consumption is important in this relationship. The association between education level and dietary WF was no longer seen; hence, differences in total energy intake explained the dietary blue WF differences between the groups.

## 4 Discussion

This study provides individual-level estimates of the WF of Indian diets, finding them to have a higher blue WF than many other countries. Important differences exist between socio-demographic groups due to food consumption patterns, particularly regionally and between socio-economic groups. These contextual factors should be considered for sustainable diet recommendations and when predicting future water scenarios.

### 4.1 Dietary characteristics

The average energy intake in this study population was 2883 kcal/capita/day, with the highest proportion coming from rice and wheat. The average energy intake was greater than National Sample Survey (NSSO) estimates from a similar period, i.e. 2047 and 2021 kcal/capita/day for rural and urban adults, respectively (Deaton and Dreze, 2009). It is possible the nutrient intakes were over-estimated by the FFQ (Ebrahim et al., 2010), and the sample of IMS had a higher average socio-economic status compared to the average for India so possibly greater overall food intake. Diets were predominately lacto-vegetarian, and average fruit and vegetable consumption was sufficient relative to the WHO recommended level of > 400 g/capita/d (WHO and FAO, 2003). However, there were significant variations in the consumption of foods between socio-demographic groups, including regional differences in cereal consumption, reflecting the diversity of diets in India.

### 4.2 Green and blue water footprints of foods groups and diets in India

The mean green WF of diets in this Indian population was 2531 l/capita/day, with the highest contributor being vegetable oils (19%). The mean blue WF was 737 l/capita/day with the greatest contribution from wheat (31%). There are currently no comparable middle-income country data available, although the combined green and blue WF (3268 l/capita/day) is similar to a South European vegetarian diet (3176 l/capita/day), lower than the average South European diet (5364 l/capita/day) but much higher than other estimates for European diets (Vanham et al., 2013). The mean blue WF was considerably greater than that found for an EU reference diet (299 l/capita/day) (Vanham et al., 2013), and for a UK diet (160 l/capita/day) (Hess et al., 2015). The differences in dietary WF can be attributed to consumption patterns (e.g. greater meat consumption in typical South European diets) and variation in the WFs of food items due to climate and yield at the location of production (Blas et al., 2016). Importantly, the high dietary blue WF demonstrates the dependency of Indian diets on ground- and surface-water resources. Increased irrigation coverage helped spur rapid growth in agricultural production during India's Green Revolution and has been a key factor underlying India's self-sufficiency in grain production (Hira, 2009, Pingali, 2012). Water scarcity is now becoming an increasing concern, with evidence that the water table is falling (Central Ground Water Board, Ministry of Water Resources, Govt of India, 2016) and the Ganges aquifer is being depleted at rapid rates (Rodell et al., 2009. Tiwari et al., 2009). However, recent findings from high-resolution in situ records of groundwater levels in the Indo-Gangetic basin suggest that the severity of depletion is very localised and water quality may be a greater concern, including arsenic and salt contamination (MacDonald et al., 2016). Nevertheless, given projected population growth and the current water yield gap in Indian agriculture, water demand is likely to increase (Jägermeyr et al., 2016). Additionally, for the localised areas where groundwater tables are rapidly falling, farmers face greater costs to extract irrigation water (Ahmed et al., 2014, Sekhri, 2013). Therefore, the high dependency of Indian diets on blue water has implications for economic, social and environmental sustainability.

In this study, the foods with the highest WF per g and per kcal were poultry products, which relates to the WF of feed and the feed conversion efficiency. When considered per g, the foods with the lowest WFs were fruits, which is consistent with global averages (Mekonnen and Hoekstra, 2011). However, when considered per calorie, the WF of fruit and vegetables increased relative to other food items, which is again consistent with global estimates. This highlights the importance of considering both indicators assessing the impact of sustainable diet recommendations, as both can be useful from economic and health perspectives (Masset et al., 2015).

Importantly, there are large variations in the WF of crops and their relative blue or green water use due to climate and agricultural practices. For example, paddy rice has a high green WF as it is grown in the *Kharif* (monsoon) season with higher water availability, while wheat is grown in the *Rabi* season when irrigation (blue water) substitutes the lack of rainfall (Timsina and Connor, 2001). Phenological characteristics also determine WFs, for example, wheat demands a cool and wet period in the growing season and dry climate for ripening and hence may not be suited to the *Kharif* season. In terms of water scarcity, these differences could mean that foods with a higher green WF (e.g. rice) may be more susceptible to erratic rainfall, whereas those with a higher blue WF (e.g. wheat) may contribute to groundwater depletion and be susceptible to groundwater scarcity.

### 4.3 Associations of dietary blue water footprint with socio-demographic characteristics

Socio-demographic groups were significantly associated with dietary blue WF, due to the amount and type of food consumed. Region was the strongest predictor of dietary blue WF, which is important to consider in terms of India's current water situation. The blue WF of diets in the north was ~ 1.5-fold greater than those in the South, i.e. 846 l/capita/day compared to 611 l/capita/day, and groundwater scarcity is most widespread in the north of India. Dietary change could be an important adaptation to limited groundwater resources, for example substituting wheat and rice by other cereal crops with lower water demand (e.g. sorghum and millet).

Associations between dietary blue WF and other socio-demographic factors were less pronounced than those for region, but still significant. Females and older participants had a lower dietary blue WF, mostly due to lower total energy intake. Hindus consumed less animal-source foods and their diets had lower blue WFs. Urban diets had a greater blue WF than more traditional, rural diets, even when accounting for total energy, and dietary blue WF increased with greater standard of living. Previously, urbanisation and increasing socio-economic status has been linked to the nutrition transition in India (Bowen et al., 2011, Shetty, 2002). This is typified by increasing consumption of animal products (especially dairy and poultry), vegetable oils and processed foods, with lower consumption of cereals and pulses (Misra et al., 2011). This study suggests changing diets towards increased total energy intake and consumption of animal products, as in many western countries, may increase dietary blue WFs with implications for water stress.

### 4.4 Study limitations

The dietary intake data are likely to include inaccuracies as FFQs can introduce measurement error and over-estimate food consumption (Bowen et al., 2012). Previous studies have used food supply data from Food Balance Sheets as a proxy for dietary intake (Hess et al., 2015, Vanham et al., 2013), yet these data are also subject to substantial error (Serra-Majem et al., 2003). An advantage of individual-level dietary intake data is that inter-individual variation in dietary WFs can be quantified and analysed with respect to socioeconomic, cultural and demographic factors.

The WF estimates for the food groups are subject to assumptions that will have introduced measurement error. The majority of participants were from urban areas so their location of residency was considered a poor predictor of the location of food production. Cereal grains, for example, are traded across long distances in India including via the nationally-coordinated government procurement, storage and redistribution activities (Department of Food and Public Distribution, India, 2016). Reliable spatial integration of production and consumption data would require substantial further analysis and modelling which was considered outside the scope of this study. Therefore, we estimated average WFs from state-level values to quantify the uncertainty of WF estimates, although this would still underestimate full variability of green and blue WFs due to local environmental factors and production systems. Future research could account for transport of food items and use more spatially-refined WF estimates. Additionally, specific data were not available from the WF Network for some food items so a substitute was used, or it was not considered in the analysis. Most of these items fell in the “other” category, and substantial effort was made to match as many as possible (Appendix Table A.1). The WF of fish was based on carp feed components and a feed conversion ratio (Suresh, 2007), but did not consider the prevalence of different species, production systems or the type of feed used. Nevertheless, fish consumption was relatively low in this study population so the effect on dietary WFs would be marginal.

Finally, these findings are not fully generalizable to India. The IMS survey was not intended to be nationally representative, but to assess the effects of rural-to-urban migration; therefore, the sample includes a greater proportion of urban dwellers than is found across India. Additionally, we have mainly focused on blue WF, which although particularly relevant for India due to high irrigation use and water scarcity concerns, does not capture relative green water use.

### 4.5 Study relevance and future research

This study provides an insight into the dependency of Indian diets on water resources, finding greater blue WFs than previous estimates from high income countries. These dietary WF estimates can be used to assess potential water scenarios as food consumption patterns change. Dietary blue WF varies significantly between regions and future investigation should focus on improving our understanding of the water demands of local diets. Analysis at refined geographical and social levels will help inform policy by identifying realistic dietary changes based on culturally acceptable limits, as well as providing evidence to effectively target interventions and incorporate sustainable diet recommendations into existing nutrition programmes.

Additionally, it is possible to consider the impact of blue water use at the local level using Life Cycle Assessment-based methods that include relative blue water scarcity (Hess et al., 2016, Ridoutt et al., 2012, Ridoutt et al., 2009). Future analyses could also capture green WF and compare this with local water availability from precipitation and potential yields (Hoekstra, 2016). This could inform optimisation of production location to suit local climatic and environmental factors, although this approach would require supportive international and interstate trade policies (Dalin et al., 2012). Potential environmental changes, e.g. altered precipitation patterns, may also need consideration.

Linking diets and WFs demonstrates the dependence of our food systems on water resources, and frames understanding from a consumer point of view, which could encourage behaviour change (Vanclay et al., 2011). It will also aid analysis of future water requirements as diets change. Other environmental, social and economic factors can be incorporated with WF measures using optimisation modelling to analyse co-benefits and trade-offs, and give acceptable, contextually appropriate, and realistic sustainable dietary recommendations.

## Figures and Tables

**Table 1:** Descriptive characteristics of the study sample (N = 6775).

Socio-demographic characteristic	n (%)
Total	6775
**Gender**	
Female	2895 (42.7)
Male	3880 (57.3)
**Region**	
North	2036 (30.1)
East	125 (1.9)
South	3292 (48.6)
West	1322 (19.5)
**Residency**	
Rural	2976 (44.0)
Urban	3796 (56.0)
**Religion**	
Hindu	6174 (91.1)
Other	601 (8.9)
**Formal education**	
None	766 (11.3)
Primary school	912 (13.5)
Secondary school	3248 (47.9)
Tertiary education	1849 (27.3)
**Married**	
Yes	5944 (87.7)
No	831 (12.3)
**Occupation**	
Unemployed	2571 (38.0)
Manual	1155 (17.1)
Skilled manual	1436 (21.2)
Non-manual	1128 (16.7)
Professional	485 (7.2)
**Standard of living index tertiles**	
Low	2508 (37.0)
Middle	2496 (36.8)
High	1771 (26.1)
**Household owning agricultural land**	
Yes	2694 (39.8)
No	4081 (60.2)

**Table 2:** Mean consumption of food categories by socio-demographic group (n = 6775). Standard deviation in brackets.

Socio-demographic characteristic	Mean consumption of food group (g/capita/day)					
	Rice	Wheat	Meat	Dairy and eggs	Fruit and vegetables	Vegetable oils
Total population	169 (106)	182 (141)	21 (35)	354 (211)	392 (204)	53 (25)

Gender						
Female	159 (91)	155 (119)	18 (30)	324 (191)	407 (192)	45 (21)
Male	176 (116)	202 (152)	23 (39)	376 (222)	439 (211)	53 (24)

Region						
North	74 (48)	281 (121)	10 (33)	430 (229)	402 (193)	49 (18)
South	229 (100)	71 (47)	23 (32)	326 (255)	374 (210)	59 (26)
East	148 (111)	225 (131)	30 (38)	355 (201)	346 (176)	39 (18)
West	165 (74)	302 (109)	14 (23)	236 (139)	432 (199)	70 (26)

Religion						
Hindu	168 (106)	183 (142)	19 (34)	353 (211)	391 (203)	49 (23)
Other	177 (110)	172 (125)	39 (42)	358 (215)	413 (212)	50 (21)

SLI tertiles						
Low	185 (113)	159 (139)	19 (30)	291 (189)	319 (181)	47 (26)
Middle	182 (99)	173 (133)	24 (34)	361 (208)	423 (201)	51 (23)
High	127 (95)	228 (143)	20 (42)	431 (219)	452 (207)	49 (19)

**Table 3:** Average water footprint (WF) characteristics of diets in the study population, including the top five contributing food categories to blue WF. The standard deviation takes into account inter-individual variation in consumption data but assumes no within-food group variation in WF.

Water footprint	Mean (SD) (l/capita/day)	Proportion of water footprint from (%)				
		Wheat	Rice	Dairy and eggs	Fruit and vegetables	Vegetable oils
Blue	737 (263)	30.9	18.7	17.3	9.6	8.8
Green	2531 (885)	7.4	14.7	15.6	9.7	18.4

**Table 4:** Results from mixed effects linear regression of socio-demographic characteristics and blue water footprint (n = 6775). Statistical significance shown by * for < 0.05, ** for < 0.01, and *** for < 0.001.

Variable	Mean blue WF (SD) (l/capita/day)	Unadjusted R (95% CI)	Adjusted R (95% CI)^a^	Energy adjusted R (95% CI)^b^
Age	737 (263)	− 3.32 (− 3.97 to − 2.67^)⁎⁎⁎^	− 3.01 (− 3.63 to − 2.39)^⁎⁎⁎^	− 0.502 (− 0.777 to − 0.277)^⁎⁎⁎^
Gender				
Male	796 (275)	Reference		
Female	658 (223)	− 131 (− 142 to − 121)^⁎⁎⁎^	− 140 (− 130 to − 151)^⁎⁎⁎^	− 19.3 (− 14.5 to − 24.1)^⁎⁎⁎^
Region				
South	611 (206)	Reference		
North	846 (262)	237 (223 to 252)^⁎⁎⁎^	193 (178 to 207)^⁎⁎⁎^	110 (104 to 117)^⁎⁎⁎^
East	824 (297)	198 (156 to 240)^⁎⁎⁎^	189 (150 to 228)^⁎⁎⁎^	92.7 (75.5 to 110)^⁎⁎⁎^
West	877 (238)	262 (245 to 279)^⁎⁎⁎^	212 (194 to 230)^⁎⁎⁎^	65.6 (57.2 to 73.9)^⁎⁎⁎^
Residency				
Urban	781 (255)	Reference		
Rural	663 (260)	− 103 (− 115 to − 92)^⁎⁎⁎^	− 82.9 (− 95.3 to − 70.4)^⁎⁎⁎^	− 33.3 (− 38.7 to − 27.9)^⁎⁎⁎^
Religion				
Hindu	734 (263)	Reference		
Other	768 (260)	32.5 (5.95 to 59.1)^⁎^	38.2 (18.2 to 58.3)^⁎⁎⁎^	36.0 (26.9 to 45.0)^⁎⁎⁎^
Education				
No education	540 (231)	Reference		
Primary	635 (221)	94.0 (71.1 to 117)^⁎⁎⁎^	44.5 (24.1 to 64.9)^⁎⁎⁎^	6.89 (− 1.96 to 15.7)
Secondary	768 (256)	202 (182 to 221)^⁎⁎⁎^	53.6 (34.9 to 72.4)^⁎⁎⁎^	6.14 (− 2.02 to 14.3)
Tertiary	815 (251)	241 (219 to 262)^⁎⁎⁎^	58.5 (37.7 to 79.2)^⁎⁎⁎^	6.93 (− 2.14 to 16.0)
SLI tertiles				
Low	676 (269)	Reference		
Middle	743 (243)	71.3 (58.2 to 84.5)^⁎⁎⁎^	51.8 (38.6 to 65.0)^⁎⁎⁎^	12.8 (7.06 to 18.5)^⁎⁎⁎^
High	816(258)	143 (128 to 158)^⁎⁎⁎^	92.8 (76.7 to 109)^⁎⁎⁎^	29.4 (22.4 to 36.4)^⁎⁎⁎^

a Adjusted for gender, age, region, SLI index, residency (rural/urban), education, occupation, religion.

b Adjusted for total energy intake, gender, age, region, SLI index, residency (rural/urban), education, occupation, religion.

## Appendix A Supplementary data

Supplementary tables Image 1
