# Corrigendum to “The water use of Indian diets and socio-demographic factors related to dietary blue water footprint” [Sci. Total Environ. 587–588 (2017) 128–136]

**DOI:** 10.1016/j.scitotenv.2019.01.203

**Published:** 2019-04-15

**Authors:** Francesca Harris, Rosemary F. Green, Edward J.M. Joy, Benjamin Kayatz, Andy Haines, Alan D. Dangour

**Affiliations:** a Department of Population Health, London School of Hygiene & Tropical Medicine, Keppel Street, London WC1E 7HT, UK; b Leverhulme Centre for Integrative Research on Agriculture and Health (LCIRAH), 36 Gordon Square, London WC1H 0PD, UK; c GFZ German Research Centre for Geosciences, Telegrafenberg, 14473 Potsdam, Germany; d Department of Social and Environmental Health Research, London School of Hygiene & Tropical Medicine, 15-17 Tavistock Place, London WC1H 9SH, UK

The authors regret that there is an error in Fig. 3, panel b. The incorrect map was used for the green water footprint of rice in Indian states. Please see the correct figure below:

**Fig. 3:**
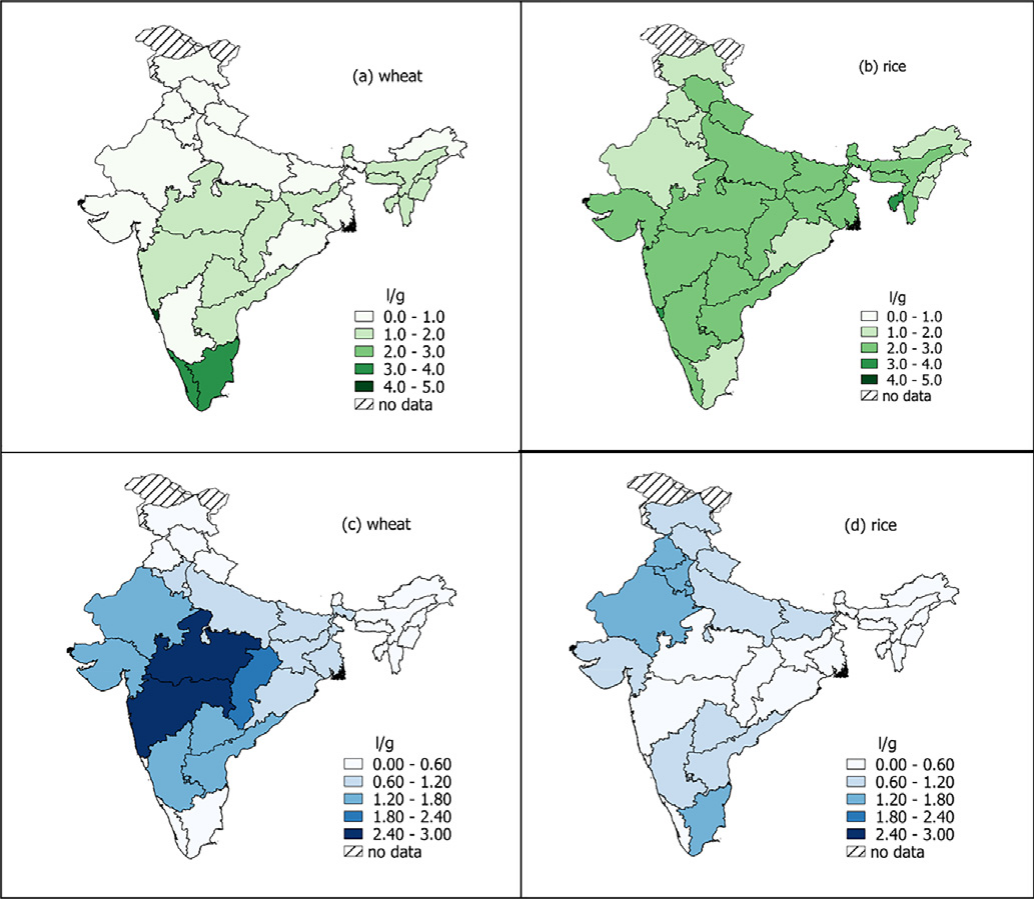
The green and blue water footprints of wheat (panels a and c) and rice (panels b and d) by state in India. Water footprint data are from the Water Footprint Network (Mekonnen and Hoekstra, 2011); boundary polygons were downloaded from the GADM database of Global Administrative Areas (version 2.8, http://www.gadm.org/) and Natural Earth Data (http://www.naturalearthdata.com). Mapping software: QGIS version 2.20.1.

All other figures and results were accurate in the original paper and were not affected by this correction. The authors would like to apologise for any inconvenience caused.

