# Supplementary material for: The water use of Indian diets and socio-demographic factors related to dietary blue water footprint
Source: Sci Total Environ. 2017 Jun 1;587-588:128–36. doi: 10.1016/j.scitotenv.2017.02.085 (PMC5378197; doi:10.1016/j.scitotenv.2017.02.085)
Supplement: Supplementary file 1 — Supplementary tables [file mmc1.docx]

**Appendix – Supplementary Tables**

**Table A.1 The 199 Food ingredients used in the Indian Migration Study, their allocated food group and matched water footprint estimation method**

| **IMS Food Item** | **Water Footprint**  **Network name** | **Water footprint (blue**  **/green)** | **Estimation**  **1=genuine**  **2=imputed**  **3=average of the food group**  **4= not available** | **Allocated food group** |  |
| --- | --- | --- | --- | --- | --- |
| Bajra (pearl millet) | Millet | Green | 3 | Cereals - other |  |
| Bajra (pearl millet) | Millet | Blue | 3 | Cereals - other |  |
| Bajre ki atta | Millet | Green | 3 | Cereals - other |  |
| Bajre ki atta | Millet | Blue | 3 | Cereals - other |  |
| Bhagar (wild grass seed) | Millet | Green | 3 | Cereals - other |  |
| Bhagar (wild grass seed) | Millet | Blue | 3 | Cereals - other |  |
| Corn flour | Maize (corn) flour | Green | 1 | Cereals - other |  |
| Corn flour | Maize (corn) flour | Blue | 1 | Cereals - other |  |
| Cornflakes | Maize (corn) nes | Green | 2 | Cereals - other |  |
| Cornflakes | Maize (corn) nes | Blue | 2 | Cereals - other |  |
| Jowar (sorghum) flour | Grain sorghum | Green | 1 | Cereals - other |  |
| Jowar (sorghum) flour | Grain sorghum | Blue | 1 | Cereals - other |  |
| Ragi (millet) | Millet | Green | 1 | Cereals - other |  |
| Ragi (millet) | Millet | Blue | 1 | Cereals - other |  |
| Ragi flour | Millet | Green | 3 | Cereals - other |  |
| Ragi flour | Millet | Blue | 3 | Cereals - other |  |
| Puffed rice | Rice, semi-milled or wholly milled, whether or not polished or glazed | Green | 3 | Cereals - rice |  |
| Puffed rice | Rice, semi-milled or wholly milled, whether or not polished or glazed | Blue | 3 | Cereals - rice |  |
| Rice (polished ) | Rice, semi-milled or wholly milled, whether or not polished or glazed | Green | 1 | Cereals - rice |  |
| Rice (polished ) | Rice, semi-milled or wholly milled, whether or not polished or glazed | Blue | 1 | Cereals - rice |  |
| Rice flakes | Rice, semi-milled or wholly milled, whether or not polished or glazed | Green | 3 | Cereals - rice |  |
| Rice flakes | Rice, semi-milled or wholly milled, whether or not polished or glazed | Blue | 3 | Cereals - rice |  |
| Rice flour | Rice flour | Green | 1 | Cereals - rice |  |
| Rice flour | Rice flour | Blue | 1 | Cereals - rice |  |
| bread | Wheat bread | Green | 1 | Cereals - wheat |  |
| bread | Wheat bread | Blue | 1 | Cereals - wheat |  |
| Dalia | Wheat (Durum wheat, Wheat nes and meslin) | Green | 1 | Cereals - wheat |  |
| Dalia | Wheat (Durum wheat, Wheat nes and meslin) | Blue | 1 | Cereals - wheat |  |
| Noodles | Dry pasta | Green | 2 | Cereals - wheat |  |
| Noodles | Dry pasta | Blue | 2 | Cereals - wheat |  |
| Rava | Wheat (Durum wheat, Wheat nes and meslin) | Green | 1 | Cereals - wheat |  |
| Rava | Wheat (Durum wheat, Wheat nes and meslin) | Blue | 1 | Cereals - wheat |  |
| Wheat flour | Wheat or meslin flour | Green | 1 | Cereals - wheat |  |
| Wheat flour | Wheat or meslin flour | Blue | 1 | Cereals - wheat |  |
| Butter | Butter | Green | 1 | Dairy - butter/ghee |  |
| Butter | Butter | Blue | 1 | Dairy - butter/ghee |  |
| Ghee | Butter | Green | 1 | Dairy - butter/ghee |  |
| Ghee | Butter | Blue | 1 | Dairy - butter/ghee |  |
| Cream | Milk and cream not concentrated and unsweetened exceeding 6% fat | Green | 1 | Dairy - hi-fat |  |
| Cream | Milk and cream not concentrated and unsweetened exceeding 6% fat | Blue | 1 | Dairy - hi-fat |  |
| Ice cream | Milk and cream nes sweetened | Green | 3 | Dairy - hi-fat |  |
| Ice cream | Milk and cream nes sweetened | Blue | 3 | Dairy - hi-fat |  |
| Khoa | Milk and cream powder sweetened exceeding 1.5% fat | Green | 1 | Dairy - hi-fat |  |
| Khoa | Milk and cream powder sweetened exceeding 1.5% fat | Blue | 1 | Dairy - hi-fat |  |
| Paneer | Cheese, fresh (including whey cheese) unfermented, and curd | Green | 1 | Dairy - hi-fat |  |
| Paneer | Cheese, fresh (including whey cheese) unfermented, and curd | Blue | 1 | Dairy - hi-fat |  |
| Curds | Buttermilk,curdled milk & cream,kephir & ferm or acid milk & cream nes | Green | 1 | Dairy - lo-fat |  |
| Curds | Buttermilk,curdled milk & cream,kephir & ferm or acid milk & cream nes | Blue | 1 | Dairy - lo-fat |  |
| Hung curd | Buttermilk,curdled milk & cream,kephir & ferm or acid milk & cream nes | Green | 1 | Dairy - lo-fat |  |
| Hung curd | Buttermilk,curdled milk & cream,kephir & ferm or acid milk & cream nes | Blue | 1 | Dairy - lo-fat |  |
| Milk | Milk not concentrated & unsweetened exceeding 1% not exceeding 6% fat | Green | 1 | Dairy - lo-fat |  |
| Milk | Milk not concentrated & unsweetened exceeding 1% not exceeding 6% fat | Blue | 1 | Dairy - lo-fat |  |
| Egg | Eggs, bird, not in shell nes | Green | 1 | Egg |  |
| Egg | Eggs, bird, not in shell nes | Blue | 1 | Egg |  |
| Fish | N/A | Green | 2 | Fish, seafood | |
| Fish | N/A | Blue | 2 | Fish, seafood | |
| Prawn | N/A | Green | 2 | Fish, seafood | |
| Prawn | N/A | Blue | 2 |  | |
| Banana | Bananas including plantains, fresh or dried | Green | 1 | Fruit - banana |  |
| Banana | Bananas including plantains, fresh or dried | Blue | 1 | Fruit - banana |  |
| Grapes | Grapes, fresh | Green | 1 | Fruit - grapes |  |
| Grapes | Berries Nes | Blue | 2 | Fruit - grapes |  |
| Guava | Guavas, mangoes and mangosteens, fresh or dried | Green | 1 | Fruit - guava |  |
| Guava | Guavas, mangoes and mangosteens, fresh or dried | Blue | 1 | Fruit - guava |  |
| Raw mango | Guavas, mangoes and mangosteens, fresh or dried | Green | 1 | Fruit - mango |  |
| Raw mango | Guavas, mangoes and mangosteens, fresh or dried | Blue | 1 | Fruit - mango |  |
| Musk melon | Other melons | Green | 3 | Fruit - melon |  |
| Musk melon | Other melons | Blue | 3 | Fruit - melon |  |
| Watermelon | Watermelons | Green | 1 | Fruit - melon |  |
| Watermelon | Watermelons | Blue | 1 | Fruit - melon |  |
| Orange | Oranges, fresh or dried | Green | 1 | Fruit - orange |  |
| Orange | Oranges, fresh or dried | Blue | 1 | Fruit - orange |  |
| Amla | Berries Nes | Green | 3 | Fruit - other |  |
| Amla | Berries Nes | Blue | 3 | Fruit - other |  |
| Apple | Apples, fresh | Green | 1 | Fruit - other |  |
| Apple | Apples, fresh | Blue | 1 | Fruit - other |  |
| Coconut | Coconut | Green | 1 | Fruit - other |  |
| Coconut | Coconut | Blue | 1 | Fruit - other |  |
| Coconut milk | Coconut | Green | 2 | Fruit - other |  |
| Coconut milk | Coconut | Blue | 2 | Fruit - other |  |
| Copra | Copra | Green | 1 | Fruit - other |  |
| Copra | Copra | Blue | 1 | Fruit - other |  |
| Custard apple | Apples, fresh | Green | 1 | Fruit - other |  |
| Custard apple | Apples, fresh | Blue | 1 | Fruit - other |  |
| Dry mango slice | Guavas, mangoes and mangosteens, fresh or dried | Green | 1 | Fruit - other |  |
| Dry mango slice | Guavas, mangoes and mangosteens, fresh or dried | Blue | 1 | Fruit - other |  |
| Jack fruit | Fruits, fresh nes | Green | 3 | Fruit - other |  |
| Jack fruit | Fruits, fresh nes | Blue | 3 | Fruit - other |  |
| Jamoon | Plums and sloes, fresh | Green | 2 | Fruit - other |  |
| Jamoon | Plums and sloes, fresh | Blue | 2 | Fruit - other |  |
| Kiwi | Fruits, fresh nes | Green | 3 | Fruit - other |  |
| Kiwi | Fruits, fresh nes | Blue | 3 | Fruit - other |  |
| Lemon | Lemons and limes, fresh or dried | Green | 1 | Fruit - other |  |
| Lemon | Oranges, fresh or dried | Blue | 2 | Fruit - other |  |
| Lemon juice | Citrus fruit juice nes exc mx unferment unspiritd,wthr/nt sug/sweet | Green | 1 | Fruit - other |  |
| Lemon juice | Oranges, fresh or dried | Blue | 2 | Fruit - other |  |
| Lime juice | Citrus fruit juice nes exc mx unferment unspiritd,wthr/nt sug/sweet | Green | 1 | Fruit - other |  |
| Lime juice | Oranges, fresh or dried | Blue | 2 | Fruit - other |  |
| Litchis | Fruits, fresh nes | Green | 3 | Fruit - other |  |
| Litchis | Fruits, fresh nes | Blue | 3 | Fruit - other |  |
| Palmyra | Fruits, fresh nes | Green | 3 | Fruit - other |  |
| Palmyra | Fruits, fresh nes | Blue | 3 | Fruit - other |  |
| Peaches | Peaches, including nectarines, fresh | Green | 1 | Fruit - other |  |
| Peaches | Peaches, including nectarines, fresh | Blue | 1 | Fruit - other |  |
| Pears | Pears and quinces, fresh | Green | 1 | Fruit - other |  |
| Pears | Pears and quinces, fresh | Blue | 1 | Fruit - other |  |
| Pineapple | Pineapples, fresh or dried | Green | 1 | Fruit - other |  |
| Pineapple | Pineapples, fresh or dried | Blue | 1 | Fruit - other |  |
| Plums | Plums and sloes, fresh | Green | 1 | Fruit - other |  |
| Plums | Plums and sloes, fresh | Blue | 1 | Fruit - other |  |
| Pomegranate | Fruits, fresh nes | Green | 3 | Fruit - other |  |
| Pomegranate | Fruits, fresh nes | Blue | 3 | Fruit - other |  |
| Raisins | Grapes, dried | Green | 2 | Fruit - other |  |
| Raisins | Berries Nes | Blue | 2 | Fruit - other |  |
| Raw plantain | Bananas including plantains, fresh or dried | Green | 1 | Fruit - other |  |
| Raw plantain | Bananas including plantains, fresh or dried | Blue | 1 | Fruit - other |  |
| Sapota | Stone fruit, nes | Green | 2 | Fruit - other |  |
| Sapota | Stone fruit, nes | Blue | 2 | Fruit - other |  |
| Sweet lime | Lemons and limes, fresh or dried | Green | 1 | Fruit - other |  |
| Sweet lime | Oranges, fresh or dried | Blue | 2 | Fruit - other |  |
| Tamarind | Fruits, fresh nes | Green | 3 | Fruit - other |  |
| Tamarind | Fruits, fresh nes | Blue | 3 | Fruit - other |  |
| Zizyphus | Fruits, fresh nes | Green | 3 | Fruit - other |  |
| Zizyphus | Fruits, fresh nes | Blue | 3 | Fruit - other |  |
| Papaya | Papaws (papayas), fresh | Green | 1 | Fruit - papaya |  |
| Papaya | Papaws (papayas), fresh | Blue | 1 | Fruit - papaya |  |
| Amaranth | Lettuce, fresh or chilled nes | Green | 2 | Leafy veg |  |
| Amaranth | Lettuce, fresh or chilled nes | Blue | 2 | Leafy veg |  |
| Cabbage | Cabbages,kohlrabi,kale and sim edible brassicas nes,fresh or chilled | Green | 1 | Leafy veg |  |
| Cabbage | Cabbages,kohlrabi,kale and sim edible brassicas nes,fresh or chilled | Blue | 1 | Leafy veg |  |
| Corriander leaves | Lettuce, fresh or chilled nes | Green | 2 | Leafy veg |  |
| Corriander leaves | Lettuce, fresh or chilled nes | Blue | 2 | Leafy veg |  |
| Gogu | Lettuce, fresh or chilled nes | Green | 2 | Leafy veg |  |
| Gogu | Lettuce, fresh or chilled nes | Blue | 2 | Leafy veg |  |
| Green vegetable (dhantu) | Lettuce, fresh or chilled nes | Green | 2 | Leafy veg |  |
| Green vegetable (dhantu) | Lettuce, fresh or chilled nes | Blue | 2 | Leafy veg |  |
| Mint leaves | Lettuce, fresh or chilled nes | Green | 2 | Leafy veg |  |
| Mint leaves | Lettuce, fresh or chilled nes | Blue | 2 | Leafy veg |  |
| Spinach | Lettuce, fresh or chilled nes | Green | 2 | Leafy veg |  |
| Spinach | Lettuce, fresh or chilled nes | Blue | 2 | Leafy veg |  |
| Cluster beans | Beans dried, shelled, whether or not skinned or split, nes | Green | 3 | Legumes |  |
| Cluster beans | Beans dried, shelled, whether or not skinned or split, nes | Blue | 3 | Legumes |  |
| Groundnut | Ground-nuts shelld,whether or not broken,not roastd or otherwise cookd | Green | 1 | Legumes |  |
| Groundnut | Ground-nuts shelld,whether or not broken,not roastd or otherwise cookd | Blue | 1 | Legumes |  |
| Kidney Beans | Kidney beans&white pea beans drid shelld,whether o not skinnd o split | Green | 1 | Legumes |  |
| Kidney Beans | Kidney beans&white pea beans drid shelld,whether o not skinnd o split | Blue | 1 | Legumes |  |
| Mutton | Sheep cuts, boneless, fresh or chilled | Green | 1 | Meat - mutton |  |
| Mutton | Sheep cuts, boneless, fresh or chilled | Blue | 1 | Meat - mutton |  |
| Brain | Edible offal average | Green | 2 | Meat - other |  |
| Brain | Edible offal average | Blue | 2 | Meat - other |  |
| Liver | Edible offal average | Green | 2 | Meat - other |  |
| Liver | Edible offal average | Blue | 2 | Meat - other |  |
| Pigeon | N/A | Green | 4 | Meat - other |  |
| Pigeon | N/A | Blue | 4 | Meat - other |  |
| Rabbit | N/A | Green | 4 | Meat - other |  |
| Rabbit | N/A | Blue | 4 | Meat - other |  |
| Salami | Swine meat cured, nes | Green | 1 | Meat - other |  |
| Salami | Swine meat cured, nes | Blue | 1 | Meat - other |  |
| Chicken | Dom fowl,duck,goose&guinea fowl meat&meat offal prep/presvd exc livers | Green | 2 | Meat - poultry |  |
| Chicken | Dom fowl,duck,goose&guinea fowl meat&meat offal prep/presvd exc livers | Blue | 2 | Meat - poultry |  |
| Other poultry | Dom fowl,duck,goose&guinea fowl meat&meat offal prep/presvd exc livers | Green | 4 | Meat - poultry |  |
| Other poultry | Dom fowl,duck,goose&guinea fowl meat&meat offal prep/presvd exc livers | Blue | 4 | Meat - poultry |  |
| Almonds | Peaches, including nectarines, fresh | Green | 2 | Nuts and seeds |  |
| Almonds | Peaches, including nectarines, fresh | Blue | 2 | Nuts and seeds |  |
| Cashewnuts | Cashew nut | Green | 1 | Nuts and seeds |  |
| Cashewnuts | Cashew nut | Blue | 1 | Nuts and seeds |  |
| Chironji | Guavas, mangoes and mangosteens, fresh or dried | Green | 2 | Nuts and seeds |  |
| Chironji | Guavas, mangoes and mangosteens, fresh or dried | Blue | 2 | Nuts and seeds |  |
| Pistachio nut | Guavas, mangoes and mangosteens, fresh or dried | Green | 2 | Nuts and seeds |  |
| Pistachio nut | Guavas, mangoes and mangosteens, fresh or dried | Blue | 2 | Nuts and seeds |  |
| Sesame seeds | Sesamum seeds, whether or not broken | Green | 1 | Nuts and seeds |  |
| Sesame seeds | Sesamum seeds, whether or not broken | Blue | 1 | Nuts and seeds |  |
| Baking powder | N/A | Green | 4 | Other |  |
| Baking powder | N/A | Blue | 4 | Other |  |
| Beer | Beer made from malt | Green | 1 | Other |  |
| Beer | Beer made from malt | Blue | 1 | Other |  |
| Chocolate | Chocolate | Green | 1 | Other |  |
| Chocolate | Chocolate | Blue | 1 | Other |  |
| Coca-cola | N/A | Green | 4 | Other |  |
| Coca-cola | N/A | Blue | 4 | Other |  |
| Coffee powder | Coffee, roasted, not decaffeinated | Green | 2 | Other |  |
| Coffee powder | Coffee, roasted, not decaffeinated | Blue | 2 | Other |  |
| Custard powder\corn flour | Maize (corn) flour | Green | 1 | Other |  |
| Custard powder\corn flour | Maize (corn) flour | Blue | 1 | Other |  |
| Horlicks | N/A | Green | 4 | Other |  |
| Horlicks | N/A | Blue | 4 | Other |  |
| Jam | Fruits, fresh nes | Green | 3 | Other |  |
| Jam | Fruits, fresh nes | Blue | 3 | Other |  |
| Kala namak | N/A | Green | 4 | Other |  |
| Kala namak | N/A | Blue | 4 | Other |  |
| Ketchup, tomato sauce | Tomato ketchup | Green | 1 | Other |  |
| Ketchup, tomato sauce | Tomato ketchup | Blue | 1 | Other |  |
| Lemon Pickle masala | Lemons and limes, fresh or dried | Green | 2 | Other |  |
| Lemon Pickle masala | Oranges, fresh or dried | Blue | 2 | Other |  |
| Local arrack/toddy | N/A | Green | 4 | Other |  |
| Local arrack/toddy | N/A | Blue | 4 | Other |  |
| Mango pickle masala | Guavas, mangoes and mangosteens, fresh or dried | Green | 2 | Other |  |
| Mango pickle masala | Guavas, mangoes and mangosteens, fresh or dried | Blue | 2 | Other |  |
| Papad khar | N/A | Green | 4 | Other |  |
| Papad khar | N/A | Blue | 4 | Other |  |
| Pav | N/A | Green | 4 | Other |  |
| Pav | N/A | Blue | 4 | Other |  |
| Pizza | Wheat bread | Green | 2 | Other |  |
| Pizza | Wheat bread | Blue | 2 | Other |  |
| Sago | Tapioca of cassava | Green | 2 | Other |  |
| Sago | Tapioca of cassava | Blue | 2 | Other |  |
| Soda | N/A | Green | 4 | Other |  |
| Soda | N/A | Blue | 4 | Other |  |
| Soya sauce | Soya sauce | Green | 1 | Other |  |
| Soya sauce | Soya sauce | Blue | 1 | Other |  |
| Spirits (whiskey, gin, rum) | Spirits obtained by distilling grape wine or grape marc | Green | 2 | Other |  |
| Spirits (whiskey, gin, rum) | Spirits obtained by distilling grape wine or grape marc | Blue | 4 | Other |  |
| Tea powder | Tea | Green | 3 | Other |  |
| Tea powder | Tea | Blue | 3 | Other |  |
| Vinegar | Fermented beverages nes (for example, cider, perry, mead, etc) | Green | 2 | Other |  |
| Vinegar | N/A | Blue | 4 | Other |  |
| Wine | Grape wines nes,incl fort&grape must,unfermntd by add alc,in ctnr > 2l | Green | 2 | Other |  |
| Wine | N/A | Blue | 4 | Other |  |
| Honey | N/A | Green | 4 | Other - sugar |  |
| Honey | N/A | Blue | 4 | Other - sugar |  |
| Jaggery | Sugar cane, fresh or dried, whether or not ground | Green | 2 | Other - sugar |  |
| Jaggery | Sugar cane, fresh or dried, whether or not ground | Blue | 2 | Other - sugar |  |
| Sugar | Refined sugar, in solid form, nes | Green | 1 | Other - sugar |  |
| Sugar | Refined sugar, in solid form, nes | Blue | 1 | Other - sugar |  |
| Sugarcane | Sugar cane, fresh or dried, whether or not ground | Green | 1 | Other - sugar |  |
| Sugarcane | Sugar cane, fresh or dried, whether or not ground | Blue | 1 | Other - sugar |  |
| Potato | Potatoes, fresh or chilled nes | Green | 1 | Potato |  |
| Potato | Potatoes, fresh or chilled nes | Blue | 1 | Potato |  |
| Bengal gram dhal | pulses nes | Green | 3 | Pulses - other |  |
| Bengal gram dhal | pulses nes | Blue | 3 | Pulses - other |  |
| Besan | pulses nes | Green | 3 | Pulses - other |  |
| Besan | pulses nes | Blue | 3 | Pulses - other |  |
| Black gram | Urd,mung,black/green gram beans drid shelld,whether/not skinnd/split | Green | 1 | Pulses - other |  |
| Black gram | Urd,mung,black/green gram beans drid shelld,whether/not skinnd/split | Blue | 1 | Pulses - other |  |
| Black gram dhal | Pulses nes | Green | 3 | Pulses - other |  |
| Black gram dhal | Pulses nes | Blue | 3 | Pulses - other |  |
| Black gram dhal flour | Flour and meal of the dried leguminous vegetables of heading No 07.13 | Green | 3 | Pulses - other |  |
| Black gram dhal flour | Flour and meal of the dried leguminous vegetables of heading No 07.14 | Blue | 3 | Pulses - other |  |
| Chick peas | Chickpeas, dried, shelled, whether or not skinned or split | Green | 1 | Pulses - other |  |
| Chick peas | Chickpeas, dried, shelled, whether or not skinned or split | Blue | 1 | Pulses - other |  |
| Green gram | Urd,mung,black/green gram beans drid shelld,whether/not skinnd/split | Green | 1 | Pulses - other |  |
| Green gram | Urd,mung,black/green gram beans drid shelld,whether/not skinnd/split | Blue | 1 | Pulses - other |  |
| Green gram dal | Pulses nes | Green | 3 | Pulses - other |  |
| Green gram dal | Pulses nes | Blue | 3 | Pulses - other |  |
| Masoor dhal | Lentils dried, shelled, whether or not skinned or split | Green | 3 | Pulses - other |  |
| Masoor dhal | Lentils dried, shelled, whether or not skinned or split | Blue | 3 | Pulses - other |  |
| Peas | Peas dried, shelled, whether or not skinned or split | Green | 1 | Pulses - other |  |
| Peas | Peas dried, shelled, whether or not skinned or split | Blue | 1 | Pulses - other |  |
| Semi falli (broad bean) | Peas dried, shelled, whether or not skinned or split | Green | 3 | Pulses - other |  |
| Semi falli (broad bean) | Peas dried, shelled, whether or not skinned or split | Blue | 3 | Pulses - other |  |
| Red gram dhal | Pigeon peas | Green | 1 | Pulses - red gram |  |
| Red gram dhal | Pigeon peas | Blue | 1 | Pulses - red gram |  |
| Salt | N/A | Green | 4 | Salt |  |
| Salt | N/A | Blue | 4 | Salt |  |
| Ajwain powder | Spices nes | Green | 3 | Spices - other |  |
| Ajwain powder | Spices nes | Blue | 3 | Spices - other |  |
| Amchur powder | Spices nes | Green | 3 | Spices - other |  |
| Amchur powder | Spices nes | Blue | 3 | Spices - other |  |
| Ansi flower | Spices nes | Green | 3 | Spices - other |  |
| Ansi flower | Spices nes | Blue | 3 | Spices - other |  |
| Asofoetida | Spices nes | Green | 3 | Spices - other |  |
| Asofoetida | Spices nes | Blue | 3 | Spices - other |  |
| Bay leaves | Spices nes | Green | 3 | Spices - other |  |
| Bay leaves | Spices nes | Blue | 3 | Spices - other |  |
| Biriyani powder | Spices nes | Green | 3 | Spices - other |  |
| Biriyani powder | Spices nes | Blue | 3 | Spices - other |  |
| Black cardamom | Nutmeg, Mace, Cardamons | Green | 1 | Spices - other |  |
| Black cardamom | Nutmeg, Mace, Cardamons | Blue | 1 | Spices - other |  |
| Cardamom | Nutmeg, Mace, Cardamons | Green | 1 | Spices - other |  |
| Cardamom | Nutmeg, Mace, Cardamons | Blue | 1 | Spices - other |  |
| Cardamom powder | Nutmeg, Mace, Cardamons | Green | 1 | Spices - other |  |
| Cardamom powder | Nutmeg, Mace, Cardamons | Blue | 1 | Spices - other |  |
| Cinnamon | Spices nes | Green | 3 | Spices - other |  |
| Cinnamon | Spices nes | Blue | 3 | Spices - other |  |
| Cloves | Spices nes | Green | 3 | Spices - other |  |
| Cloves | Spices nes | Blue | 3 | Spices - other |  |
| Coriander seeds | Coriander seeds | Green | 1 | Spices - other |  |
| Coriander seeds | Coriander seeds | Blue | 1 | Spices - other |  |
| Cumin seed powder | Spices nes | Green | 3 | Spices - other |  |
| Cumin seed powder | Spices nes | Blue | 3 | Spices - other |  |
| Curry leaves | Curry | Green | 2 | Spices - other |  |
| Curry leaves | Curry | Blue | 2 | Spices - other |  |
| Danya pder | Spices nes | Green | 3 | Spices - other |  |
| Danya pder | Spices nes | Blue | 3 | Spices - other |  |
| Dry red chilly | Fruits of the genus Capsicum or Pimenta, dried, crushed or ground | Green | 1 | Spices - other |  |
| Dry red chilly | Fruits of the genus Capsicum or Pimenta, dried, crushed or ground | Blue | 1 | Spices - other |  |
| Ginger | Ginger | Green | 1 | Spices - other |  |
| Ginger | Ginger | Blue | 1 | Spices - other |  |
| Jaiphal | Spices nes | Green | 3 | Spices - other |  |
| Jaiphal | Spices nes | Blue | 3 | Spices - other |  |
| Javithri | Spices nes | Green | 3 | Spices - other |  |
| Javithri | Spices nes | Blue | 3 | Spices - other |  |
| Jeera | Spices nes | Green | 3 | Spices - other |  |
| Jeera | Spices nes | Blue | 3 | Spices - other |  |
| Kamal kakdi | Spices nes | Green | 3 | Spices - other |  |
| Kamal kakdi | Spices nes | Blue | 3 | Spices - other |  |
| Kasuri methi | Spices nes | Green | 3 | Spices - other |  |
| Kasuri methi | Spices nes | Blue | 3 | Spices - other |  |
| Methi seeds | Spices nes | Green | 3 | Spices - other |  |
| Methi seeds | Spices nes | Blue | 3 | Spices - other |  |
| Mustard | Spices nes | Green | 3 | Spices - other |  |
| Mustard | Spices nes | Blue | 3 | Spices - other |  |
| Mustard powder | Spices nes | Green | 3 | Spices - other |  |
| Mustard powder | Spices nes | Blue | 3 | Spices - other |  |
| Omum | Spices nes | Green | 3 | Spices - other |  |
| Omum | Spices nes | Blue | 3 | Spices - other |  |
| Onion seed | Spices nes | Green | 3 | Spices - other |  |
| Onion seed | Spices nes | Blue | 3 | Spices - other |  |
| Pepper corn | Pepper of the genus Piper, except cubeb pepper, crushed or ground | Green | 1 | Spices - other |  |
| Pepper corn | Pepper of the genus Piper, except cubeb pepper, crushed or ground | Blue | 1 | Spices - other |  |
| Poppy seeds | Spices nes | Green | 3 | Spices - other |  |
| Poppy seeds | Spices nes | Blue | 3 | Spices - other |  |
| Red chilly | Fruits of the genus Capsicum or Pimenta, dried, crushed or ground | Green | 1 | Spices - other |  |
| Red chilly | Fruits of the genus Capsicum or Pimenta, dried, crushed or ground | Blue | 1 | Spices - other |  |
| Red chilly powder | Fruits of the genus Capsicum or Pimenta, dried, crushed or ground | Green | 1 | Spices - other |  |
| Red chilly powder | Fruits of the genus Capsicum or Pimenta, dried, crushed or ground | Blue | 1 | Spices - other |  |
| Sambar powder | Spices nes | Green | 3 | Spices - other |  |
| Sambar powder | Spices nes | Blue | 3 | Spices - other |  |
| Saunf | Spices nes | Green | 3 | Spices - other |  |
| Saunf | Spices nes | Blue | 3 | Spices - other |  |
| Shajeera | Spices nes | Green | 3 | Spices - other |  |
| Shajeera | Spices nes | Blue | 3 | Spices - other |  |
| Turmeric pder | Turmeric (curcuma) | Green | 1 | Spices - other |  |
| Turmeric pder | Turmeric (curcuma) | Blue | 1 | Spices - other |  |
| Colacasia | Manioc (cassava), fresh or dried, whether or not sliced or pelleted | Green | 2 | Starchy roots |  |
| Colacasia | Manioc (cassava), fresh or dried, whether or not sliced or pelleted | Blue | 2 | Starchy roots |  |
| Yam | Sweet potatoes, fresh or dried, whether or not sliced or pelleted | Green | 3 | Starchy roots |  |
| Yam | Sweet potatoes, fresh or dried, whether or not sliced or pelleted | Blue | 3 | Starchy roots |  |
| Carrot | Carrots and turnips, fresh or chilled | Green | 1 | Veg - carrot |  |
| Carrot | Carrots and turnips, fresh or chilled | Blue | 1 | Veg - carrot |  |
| Bitter gourd | Pumpkins, squash and gourds | Green | 3 | Veg - gourd |  |
| Bitter gourd | Pumpkins, squash and gourds | Blue | 3 | Veg - gourd |  |
| Bottle gourd | Pumpkins, squash and gourds | Green | 3 | Veg - gourd |  |
| Bottle gourd | Pumpkins, squash and gourds | Blue | 3 | Veg - gourd |  |
| Dhemsa/tinda | Pumpkins, squash and gourds | Green | 3 | Veg - gourd |  |
| Dhemsa/tinda | Pumpkins, squash and gourds | Blue | 3 | Veg - gourd |  |
| Kundru | Pumpkins, squash and gourds | Green | 3 | Veg - gourd |  |
| Kundru | Pumpkins, squash and gourds | Blue | 3 | Veg - gourd |  |
| Lauki | Pumpkins, squash and gourds | Green | 3 | Veg - gourd |  |
| Lauki | Pumpkins, squash and gourds | Blue | 3 | Veg - gourd |  |
| Parwal | Pumpkins, squash and gourds | Green | 3 | Veg - gourd |  |
| Parwal | Pumpkins, squash and gourds | Blue | 3 | Veg - gourd |  |
| Ridge gourd | Pumpkins, squash and gourds | Green | 3 | Veg - gourd |  |
| Ridge gourd | Pumpkins, squash and gourds | Blue | 3 | Veg - gourd |  |
| Garlic | Garlic, fresh or chilled | Green | 1 | Veg - onion and garlic |  |
| Garlic | Garlic, fresh or chilled | Blue | 1 | Veg - onion and garlic |  |
| Green onion | Onions dried but not further prepared | Green | 2 | Veg - onion and garlic |  |
| Green onion | Onions dried but not further prepared | Blue | 2 | Veg - onion and garlic |  |
| Onion | Onions dried but not further prepared | Green | 2 | Veg - onion and garlic |  |
| Onion | Onions dried but not further prepared | Blue | 2 | Veg - onion and garlic |  |
| Spring onions | Onions dried but not further prepared | Green | 2 | Veg - onion and garlic |  |
| Spring onions | Onions dried but not further prepared | Blue | 2 | Veg - onion and garlic |  |
| Beetroot | Vegetables, fresh or chilled nes | Green | 3 | Veg - other |  |
| Beetroot | Vegetables, fresh or chilled nes | Blue | 3 | Veg - other |  |
| Brinjal | Aubergines(egg-plants), fresh or chilled | Green | 1 | Veg - other |  |
| Brinjal | Aubergines(egg-plants), fresh or chilled | Blue | 1 | Veg - other |  |
| Capsicum | Peppers of the genus Capsicum or of the genus Pimenta,fresh or chilled | Green | 1 | Veg - other |  |
| Capsicum | Peppers of the genus Capsicum or of the genus Pimenta,fresh or chilled | Blue | 1 | Veg - other |  |
| Cauliflower | Cauliflowers and headed broccoli, fresh or chilled | Green | 1 | Veg - other |  |
| Cauliflower | Cauliflowers and headed broccoli, fresh or chilled | Blue | 1 | Veg - other |  |
| Chow chow marrow | Pumpkin, squash and gourds | Green | 3 | Veg - other |  |
| Chow chow marrow | Pumpkin, squash and gourds | Blue | 3 | Veg - other |  |
| Cucumber | Cucumbers and gherkins, fresh or chilled | Green | 1 | Veg - other |  |
| Cucumber | Cucumbers and gherkins, fresh or chilled | Blue | 1 | Veg - other |  |
| Drum stick | Vegetables, fresh or chilled nes | Green | 3 | Veg - other |  |
| Drum stick | Vegetables, fresh or chilled nes | Blue | 3 | Veg - other |  |
| Green beans | Beans, shelled or unshelled, fresh or chilled | Green | 3 | Veg - other |  |
| Green beans | Beans, shelled or unshelled, fresh or chilled | Blue | 3 | Veg - other |  |
| Green chilli | Fruits of the genus Capsicum or Pimenta, dried, crushed or ground | Green | 1 | Veg - other |  |
| Green chilli | Fruits of the genus Capsicum or Pimenta, dried, crushed or ground | Blue | 1 | Veg - other |  |
| Ladies finger | Okra | Green | 1 | Veg - other |  |
| Ladies finger | Okra | Blue | 1 | Veg - other |  |
| Mushroom | Vegetables, fresh or chilled nes | Green | 3 | Veg - other |  |
| Mushroom | Vegetables, fresh or chilled nes | Blue | 3 | Veg - other |  |
| Radish | Vegetables, fresh or chilled nes | Green | 3 | Veg - other |  |
| Radish | Vegetables, fresh or chilled nes | Blue | 3 | Veg - other |  |
| Red pumpkin | Pumpkins, squash and gourds | Green | 3 | Veg - other |  |
| Red pumpkin | Pumpkins, squash and gourds | Blue | 3 | Veg - other |  |
| Turnip | Carrots and turnips, fresh or chilled | Green | 1 | Veg - other |  |
| Turnip | Carrots and turnips, fresh or chilled | Blue | 1 | Veg - other |  |
| Tomato puree | Tomato puree | Green | 1 | Veg - tomato |  |
| Tomato puree | Tomato puree | Blue | 1 | Veg - tomato |  |
| Tomatoes | Tomatoes, fresh or chilled | Green | 1 | Veg - tomato |  |
| Tomatoes | Tomatoes, fresh or chilled | Blue | 1 | Veg - tomato |  |
| Coconut oil | Coconut (copra) oil&its fractions refined but not chemically modified | Green | 1 | Veg oils |  |
| Coconut oil | N/A | Blue | 4 | Veg oils |  |
| Dalda | N/A | Green | 4 | Veg oils |  |
| Dalda | N/A | Blue | 4 | Veg oils |  |
| Groundnut oil | Ground-nut oil and its fractions refined but not chemically modified | Green | 1 | Veg oils |  |
| Groundnut oil | Ground-nut oil and its fractions refined but not chemically modified | Blue | 1 | Veg oils |  |
| Mustard oil | Rape,colza o mustard oil&their fract,refind but not chemically modifid | Green | 1 | Veg oils |  |
| Mustard oil | Rape,colza o mustard oil&their fract,refind but not chemically modifid | Blue | 1 | Veg oils |  |
| Palm oil | Coconut (copra) oil&its fractions refined but not chemically modified | Green | 2 | Veg oils |  |
| Palm oil | N/A | Blue | 4 | Veg oils |  |
| Soya oil | Soya-bean oil and its fractions, refined but not chemically modified | Green | 1 | Veg oils |  |
| Soya oil | Soya-bean oil and its fractions, refined but not chemically modified | Blue | 1 | Veg oils |  |
| Sunflower oil | Sunflower-sed/safflower oil&their fractions refind but nt chem modifid | Green | 1 | Veg oils |  |
| Sunflower oil | Sunflower-sed/safflower oil&their fractions refind but nt chem modifid | Blue | 1 | Veg oils |  |

| **State** | **State Size (km^2^)** |
| --- | --- |
| Andhra Pradesh | 160205 |
| Assam | 78438 |
| Chandigarh | 114 |
| Dadra and Nagar Haveli | 491 |
| Delhi | 1490 |
| Goa | 3702 |
| Gujarat | 196024 |
| Haryana | 44212 |
| Himachal Pradesh | 55673 |
| Jammu and Kashmir | 85806 |
| Maharashtra | 307713 |
| Manipur | 22327 |
| Meghalaya | 22429 |
| Karnataka | 191791 |
| Nagaland | 16579 |
| Orissa | 155707 |
| Kerala | 38863 |
| Punjab | 50362 |
| Rajasthan | 342239 |
| Tamil Nadu | 130058 |
| Tripura | 10486 |
| West Bengal | 88752 |
| Sikkim | 7096 |
| Arunachal Pradesh | 83743 |
| Mizoram | 21081 |
| Daman and Diu | 112 |
| Bihar | 94163 |
| Madhya Pradesh | 308245 |
| Uttaranchal | 53483 |
| Chhattisgarh | 135191 |
| Jharkhand | 79714 |
| Uttar Pradesh | 240928 |
| **Total Size** | **3027217** |

**Table A.2 State size in India Source: Office of Registrar General of India, Ministry of Home Affairs)**

**Table A.3 The Weighted Average Water Footprints of the 36 Food Groups, with the minimum and maximum state values for India**

| **Food group** | **Blue WF (L/kcal)**  *(min to max)* | **Green WF (L/kcal)**  *(min to max)* | **Blue WF (L/g)** *(min to max)* | **Green WF (L/g)**  *(min to max)* |
| --- | --- | --- | --- | --- |
| **Orange** | 0.01 (*0.00 to 0.03)* | 1.41 *(0.77 to 1.79)* | 0.00 *(0.00 to 0.01)* | 0.68 *(0.37 to 0.86)* |
| **Other cereals** | 0.02 (*0.00 to 0.20)* | 1.24 *(0.79 to 1.69)* | 0.07 *(0.00 to 0.69)* | 4.17 *(2.67 to 5.71)* |
| **Potato** | 0.04 *(0.00 to 0.13)* | 0.23 *(0.17 to 0.31)* | 0.04 *(0.00 to 0.12)* | 0.22 *(0.16 to 0.30)* |
| **Starchy roots** | 0.04 *(0.00 to 0.21)* | 0.55 *(0.25 to 1.02)* | 0.04 *(0.00 to 0.22)* | 0.56 *(0.26 to 1.04)* |
| **Gourd** | 0.07 *(0.00 to 0.30)* | 2.28 (1.36 to 3.17) | 0.01 *(0.00 to 0.05)* | 0.37 *(0.22 to 0.52)* |
| **Red gram** | 0.08 *(0.00 to 0.45)* | 1.51 *(1.07 to 2.33)* | 0.28 *(0.00 to 1.50)* | 5.07 *(3.59 to 7.79)* |
| **Legumes** | 0.09 *(0.00 to 0.27)* | 0.91 *(0.62 to 2.01)* | 0.45 *(0.00 to 1.40)* | 4.74 *(3.22 to 10.47)* |
| **Veg oils** | 0.10 *(0.00 to 0.47)* | 1.06 *(0.17 to 1.58)* | 0.87 *(0.00 to 4.23)* | 9.54 *(1.50 to 14.17)* |
| **Carrot** | 0.13 *(0.00 to 0.22)* | 0.17 *(0.09 to 0.35)* | 0.06 *(0.00 to 0.11)* | 0.08 *(0.04 to 0.17)* |
| **Other pulses** | 0.14 *(0.00 to 0.34)* | 1.36 *(0.81 to 1.86)* | 0.32 *(0.00 to 0.77)* | 3.11 *(1.86 to 4.24)* |
| **Leafy veg** | 0.14 *(0.00 to 0.29)* | 1.29 *(0.84 to 1.92)* | 0.05 *(0.00 to 0.09)* | 0.41 *(0.27 to 0.61)* |
| **Melon** | 0.15 *(0.00 to 0.36)* | 1.60 *(0.91 to 2.45)* | 0.02 *(0.00 to 0.06)* | 0.26 *(0.15 to 0.41)* |
| **Mutton** | 0.15 *(0.00 to 0.25)* | 2.51 *(2.34 to 9.93)* | 0.29 *(0.00 to 0.48)* | 4.87 *(4.55 to 5.69)* |
| **Spices** | 0.16 *(0.00 to 0.39)* | 1.34 *(0.82 to 1.99)* | 0.42 *(0.00 to 1.06)* | 3.63 *(2.20 to 5.37)* |
| **Other fruit** | 0.16 *(0.00 to 0.37)* | 0.87 *(0.59 to 1.20)* | 0.22 *(0.00 to 0.50)* | 1.16 *(0.79 to 1.60)* |
| **Banana** | 0.17 *(0.00 to 0.26)* | 0.23 *(0.11 to 0.72)* | 0.19 *(0.00 to 0.31)* | 0.27 *(0.13 to 0.84)* |
| **Butter/ghee** | 0.17 *(0.00 to 0.30)* | 0.49 *(0.47 to 0.61)* | 1.46 *(0.02 to 2.66)* | 4.32 *(4.11 to 5.32)* |
| **Rice** | 0.21 *(0.00 to 0.52)* | 0.60 *(0.34 to 1.12)* | 0.72 *(0.01 to 1.79)* | 2.07 *(1.19 to 3.87)* |
| **Tomato** | 0.22 *(0.00 to 0.90)* | 1.12 *(0.39 to 1.93)* | 0.04 *(0.00 to 0.18)* | 0.22 *(0.08 to 0.38)* |
| **Other meat** | 0.22 *(0.00 to 0.39)* | 2.66 *(0.24 to 3.42)* | 0.27 *(0.00 to 0.49)* | 3.32 *(3.11 to 4.28)* |
| **Nuts and seeds** | 0.26 *(0.00 to 0.75)* | 1.57 *(0.18 to 2.33)* | 1.53 *(0.00 to 4.46)* | 9.41 *(1.10 to 13.95)* |
| **Onion and garlic** | 0.26 *(0.00 to 0.45)* | 0.29 *(0.11 to 0.62)* | 0.14 *(0.00 to 0.25)* | 0.16 *(0.06 to 0.35)* |
| **Sugar** | 0.27 *(0.00 to 0.37)* | 0.25 *(0.03 to 0.64)* | 1.00 *(0.02 to 1.38)* | 0.92 *(0.13 to 2.37)* |
| **Papaya** | 0.27 *(0.00 to 0.49)* | 0.84 (*0.53 to 1.96)* | 0.09 *(0.00 to 0.16)* | 0.27 *(0.17 to 0.63)* |
| **Cream/cheese** | 0.29 *(0.00 to 0.54)* | 0.87 *(0.83 to 1.08)* | 0.92 *(0.01 to 1.67)* | 2.72 *(2.58 to 3.35)* |
| **Fish and prawns** | 0.32 *(0.00 to 0.61)* | 0.73 *(0.53 to 1.40)* | 0.29 *(0.00 to 0.57)* | 0.67 *(0.49 to 1.29)* |
| **Other** | 0.35 *(0.00 to 0.96)* | 2.12 *(1.04 to 4.86)* | 0.40 *(0.00 to 1.07)* | 2.37 *(1.16 to 5.42*) |
| **Wheat** | 0.40 *(0.00 to 0.82)* | 0.29 *(0.13 to 1.29)* | 1.37 *(0.00 to 2.82)* | 0.98 *(0.46 to 4.46)* |
| **Milk** | 0.43 *(0.01 to 0.78)* | 1.27 *(1.21 to 1.56)* | 0.29 *(0.00 to 0.52)* | 0.85 *(0.80 to 1.04)* |
| **Grapes** | 0.58 *(0.00 to 0.94)* | 0.50 *(0.37 to 0.66)* | 0.34 *(0.00 to 0.55)* | 0.29 *(0.21 to 0.38)* |
| **Mango** | 0.77 *(0.00 to 1.47)* | 1.67 *(1.21 to 3.01)* | 0.57 *(0.00 to 1.08)* | 1.24 *(0.89 to 2.23)* |
| **Other veg** | 0.84 *(0.00 to 1.82)* | 4.12 *(2.51 to 6.60)* | 0.22 *(0.00 to 0.47)* | 1.06 *(0.64 to 1.70)* |
| **Egg** | 0.96 *(0.01 to 1.96)* | 4.46 *(3.21 to 13.61)* | 1.66 *(0.01 to 3.38)* | 7.72 *(5.56 to 23.54)* |
| **Guava** | 1.11 *(0.00 to 2.13)* | 2.43 *(1.75 to 4.37)* | 0.57 *(0.00 to 1.08)* | 1.24 *(0.89 to 2.23)* |
| **Poultry** | 2.19 *(0.02 to 4.47)* | 10.19 *(7.34 to 31.07)* | 2.53 *(0.02 to 5.16)* | 11.78 *(8.49 to 35.92)* |
